# P-502. Neonatal Listeriosis in Israel 2012-2022, Clinical presentation and Outcome

**DOI:** 10.1093/ofid/ofaf695.717

**Published:** 2026-01-11

**Authors:** Galia Grisaru, Tamar Gal Tzur Yron, Nadav Michaan, Maskit Bar-Meir, Liat Ashkenazi, Michal Stein, Rima Melamed, Ronni Gur Lavy, Shadia Hassan, Shelly Lipman, Rachel Steuerman, Daniel Glikman, Dina Averbuch, Alex Guri, Diana Tasher, Tal Brosh

**Affiliations:** Tel Aviv Sourasky Medical Center, Tel Aviv, Tel Aviv, Israel; Tel Aviv Sourasky Medical Center, Tel Aviv, Tel Aviv, Israel; Tel Aviv Sourasky Medical Center, Tel Aviv, Tel Aviv, Israel; Shaare Zedek Medical Center, Jerusalem, Yerushalayim, Israel; Schneider Children’s Medical Center, Petah Tikva, HaMerkaz, Israel; Sheba Medical Center, Tel HaShomer, HaMerkaz, Israel; Soroka Medical Center, Beer Sheva, HaDarom, Israel; Shamir Medical Center, Beer Yaakov, HaMerkaz, Israel; Rambam Health Care Campus, Haifa, Hefa, Israel; Hillel Yaffe Medical Center, Hadera, HaMerkaz, Israel; Meir Medical Center, Kfar Saba, HaMerkaz, Israel; Baruch Pade (Poria) Medical Center, Tveria, HaZafon, Israel; Hadassah Medical Center, Jerusalem, Yerushalayim, Israel; Kaplan Medical Center, Rehovot, HaDarom, Israel; Pediatric Infectious Diseases Unit, Edith Wolfson Medical Center, Holon /Sackler School of Medicine, Tel-Aviv University, Holon, Tel Aviv, Israel; Assuta Ashdod Hospital, Ashdod, HaDarom, Israel

## Abstract

**Background:**

The goal of this study was to characterize the incidence clinical features, microbiologic characteristics and outcome associated with neonatal listeriosis. We also analyzed presentation, neonatal outcome at discharge, and predictors of severe presentation and outcome.

**Methods:**

We performed a multicenter retrospective observational cohort study, including all live newborns aged 0-30 days with microbiology-confirmed maternal and/or neonatal *Lm* infections, from 14 participating medical centers in Israel born between January 1, 2012, and July 1, 2022. Clinical and laboratory data were retrieved from the medical records and microbiology laboratories.

**Results:**

A total of 98 cases were included, corresponding to mean annual incidence of 7.2 per 100,000 live births. Among the neonates, 98% were classified as early onset. Preterm births accounted for 74% of cases with a median gestational age of 34 weeks. Symptomatic neonates comprised 79% of cases. Neonatal mortality reached 17%, and 21% of neonates had long-term sequels. Maternal symptoms—either flu-like or obstetric—were reported in over 60% of cases. Only 40% of mothers received antibiotic treatment ≥1 day before delivery. Of these, 33% received appropriate anti- *Lm* therapy Appropriate maternal antibiotic treatment was associated with a significant reduction in neonatal morbidity: 89% of neonates born to the untreated mothers were symptomatic versus 58% of the neonates in the treated mothers group (P=0.006).

**Conclusion:**

Neonatal listeriosis in Israel is almost always early onset and occurs within the first 2 days of life with a high case-fatality rate. In addition to gestational age at birth, another critical parameter that influenced neonatal severity was antenatal maternal antimicrobial treatment that was associated with reduced neonatal listeriosis severity. This strongly supports preemptive maternal antimicrobial therapy when maternal listeriosis is suspected. Enhanced clinical awareness and timely intervention are essential to reduce the burden of neonatal listeriosis as well as strategies to prevent pregnancy-associated listeriosis.

**Disclosures:**

All Authors: No reported disclosures

